# Effect of Collagen Types, Bacterial Strains and Storage Duration on the Quality of Probiotic Fermented Sheep’s Milk

**DOI:** 10.3390/molecules27093028

**Published:** 2022-05-08

**Authors:** Kamil Szopa, Agata Znamirowska-Piotrowska, Katarzyna Szajnar, Małgorzata Pawlos

**Affiliations:** Department of Dairy Technology, Institute of Food Technology and Nutrition, University of Rzeszow, Ćwiklińskiej 2D, 35601 Rzeszów, Poland; aznamirowska@ur.edu.pl (A.Z.-P.); kszajnar@ur.edu.pl (K.S.); mpawlos@ur.edu.pl (M.P.)

**Keywords:** bovine collagen, collagen hydrolysate, fermented sheep’s milk, probiotic

## Abstract

Collagen has become popular in dietary supplements, beverages and sports nutrition products. Therefore, the aim of this study was to evaluate the possibility of using various doses of collagen and collagen hydrolysate to produce probiotic sheep’s milk fermented with *Lactobacillus acidophilus, Lacticaseibacillus casei, Lacticaseibacillus paracasei* and *Lacticaseibacillus rhamnosus*. The effects of storage time, type and dose of collagen, and different probiotic bacteria on the physicochemical, organoleptic and microbiological properties of fermented sheep’s milk at 1 and 21 days of refrigerated storage were investigated. The addition of collagen to sheep’s milk increased the pH value after fermentation and reduced the lactic acid contents of fermented milk compared to control samples. After fermentation, the number of probiotic bacteria cells was higher than 8 log cfu g^−1^. In sheep’s milk fermented by *L. acidophilus* and *L. casei*, good survival of bacteria during storage was observed, and there was no effect of collagen dose on the growth and survival of both strains. The addition of collagen, both in the form of hydrolysate and bovine collagen, resulted in darkening of the color of the milk and increased the sweet taste intensity of the fermented sheep’s milk. However, the addition of hydrolysate was effective in reducing syneresis in each milk sample compared to its control counterpart.

## 1. Introduction

Changing lifestyles, the increase in non-communicable lifestyle diseases, growing consumer self-awareness and expectations of healthy foods have resulted in an escalating demand for functional foods. Among functional foods, milk-based foods represent almost 43% [1]. Fermented milk products (yogurt, kefir, buttermilk and probiotic milk) are very popular, and their consumption is dynamically rising. This trend is due to fermented milk’s high nutritional and dietary values and therapeutic and preventive properties [2,3,4].

The most commonly used probiotics from the lactic acid bacteria group include *Bifidobacterium animalis* ssp. *lactis*, *Lacticaseibacillus rhamnosus*, *Lactobacillus acidophilus*, *Lacticaseibacillus casei* and *Lacticaseibacillus paracasei*. To maintain their therapeutic effects, the minimum number of live probiotic cells at the time of consumption should be above 10^6^ cfu g^−1^ [5,6,7]. The beneficial effects of probiotics are variable and strain specific [8]. Probiotics could exhibit antioxidant, antimicrobial, and anti-inflammatory effects and may be used as an alternative or adjunct to antiviral therapy [9]. One of the mechanisms of health enhancement by probiotics is to stimulate the systemic immune system by inducing an immune response in the host [10,11]. This occurs due to Lactobacilli binding to specific receptors on immune cells and other tissues such as the intestinal epithelium. The receptors initiate the production of cytokines, chemokines, and T and B lymphocytes and the activation of DC dendritic cells and macrophages [8]. Another beneficial effect of probiotics is their ability to activate the mucosal immune system through the secretion of metabolites such as bacteriocins, organic acids, short-chain fatty acids and hydrogen peroxide, which exhibit antimicrobial properties [9,12]. Producing organic acids such as lactates reduces the pH value in the gut of the consumers, therefore creating unfavorable conditions for potentially pathogenic microorganisms [13]. Antimicrobial activity is also associated with the adhesion of some probiotic strains to the intestinal epithelium, thus preventing the invasion of pathogenic bacteria such as *Escherichia coli, Salmonella*, *Clostridium difficile* and *Helicobacter pylori* and resulting in nutrients’ competition [8,13]. Probiotics may also exhibit anticancer properties. Daily oral intake of microencapsulated *Lactobacillus acidophilus* resulted in significant colon tumor inhibition and reduction in tumor size. In comparison, *Lacticaseibacillus casei* has an inhibitory effect on the growth of superficial bladder and colorectal cancer [14]. *Lacticaseibacillus GG* and a variety of *L. casei ssp. rhamnosus* are the best-explored probiotics and were shown to be effective in reducing the severity and duration of diarrhoea following rotavirus infections [15]. The ingestion of probiotics helps to strengthen and maintain a well-balanced intestinal microflora. Probiotics are used to prevent and treat diseases and health disorders in people dealing with high blood pressure, high cholesterol, lactose intolerance and gastrointestinal disorders (irritable bowel syndrome, Crohn’s disease and acute diarrhoea) [13].

In addition to bovine, milk from other mammalian species like buffalo, goat and sheep is used. According to Eurostat data, sheep’s milk production in EU countries in 2019 and 2020 was 3.0 million tons [16,17]. The global production of sheep’s milk in 2019 was 10.62 million tons and accounts for 1.3% of the dairy sector, as reported by FAOSTAT [18]. Nowadays, the largest producers of sheep’s milk globally are Turkey (1.52 million tons) and China (1.17 million tons). In Poland, sheep’s milk production is 570 tons [18]. It is becoming increasingly popular as an alternative to cow’s milk-based products because of its nutritional value, compounds’ bioavailability, and an absence of allergic reactions due to the lack of the s1 fraction of casein [19,20,21,22]. Sheep’s milk is characterized by high levels of dry matter content, fat, total protein, vitamins B, C, A, D and high amounts of calcium, phosphorus, potassium, sodium, chlorine and selenium compared to cow’s milk. It is more recommended for osteoporosis patients than cow and goat milk [23]. Due to its favorable ratio of calcium and phosphorus, it is characterized by easily bioavailable calcium. It is considered a good source of the functional bioactive peptides involved in processes related to the functioning of the endocrine, digestive, immune and nervous systems. The distinct triacylglycerol structures in sheep’s milk fat have better digestibility than cow milk fat [24,25].

The application of collagen as a functional food ingredient is recommended as collagen peptides perform essential physiological functions and positively improve skin elasticity, restore lost cartilage, reduce activity-related joint pain, strengthen tendons and ligaments, and increase bone mineral density in postmenopausal women [26,27,28,29]. From the age of 25, collagen levels in the body start to decrease, which is why it is beneficial to supplement it from the age of 30. Collagen represents 30% of all human proteins and is the most important structural protein, which structure depends on its location and function in the body [30,31,32]. Currently, 29 types of collagen have been discovered, which differ in structure, properties and location in the body. It is found in the muscles, bones, skin and tendons and is the main component of connective tissue [33,34]. Collagen has become popular in dietary supplements, beverages and sports nutrition products. It is most commonly found in hydrolysate form as a dietary supplement and food additive. Collagen hydrolysates (collagen peptides) are easily absorbed and assimilated by the body. Collagen and its hydrolysates of fish, bovine and porcine origin are commonly used in the food, pharmaceutical, cosmeceutical, and nutraceutical industries [30,34].

Currently, no studies have evaluated the effects of the simultaneous addition of collagen, collagen hydrolysate and probiotics or a combination of these components on the properties of fermented sheep’s milk. Therefore, this study aimed to evaluate the possibility of using different doses of collagen hydrolysate and collagen to produce probiotic fermented sheep’s milk using *Lactobacillus acidophilus*, *Lacticaseibacillus casei*, *Lacticaseibacillus paracasei* and *Lacticaseibacillus rhamnosus*. The effects of storage time, type and dose of collagen and different types of probiotic bacteria on the physicochemical, organoleptic and microbiological properties of fermented sheep’s milk at 1 and 21 days of refrigerated storage were investigated.

## 2. Results

### 2.1. Physicochemical Properties of Fermented Milk

The results of the acidity, syneresis, color and texture of fermented sheep’s milk are presented in Table 1, Table 2, Table 3 and Table 4. The analysis of the pH value of the control (without collagen) sheep’s milk at one day of storage resulted in the lowest value in control milk fermented by *L. paracasei* (LP) and the highest value in control milk fermented by *L. casei* (LC). The addition of collagen resulted in fermented milk with a higher pH value at one day of storage than control samples without collagen. The type of collagen was found to be significant in differentiating the pH value, as milk samples with hydrolysate had a higher pH value than those with bovine collagen on day 1 of storage. Furthermore, a tendency was observed that increasing the collagen dosage from 1.5% to 3.0% resulted in mostly higher pH when both hydrolysate and bovine collagen were added.

After 21 days of cold storage, the lowest pH values were found in milk fermented by *L. paracasei* both in control milk and milk with added collagen compared to their counterparts fermented by other probiotic strains. In sheep’s milk fermented by *L. casei* after 21 days of storage, the pH value was in the range of 4.10–4.13 and showed no significant difference with respect to the type and dose of collagen. However, in sheep’s milk fermented by *L. acidophilus* LA-5, *L. paracasei* and *L. rhamnosus*, the addition of collagen to milk significantly influenced the pH value depending on the type and dose of collagen after 21 days of storage. Hence, the higher pH values were determined in the milk with hydrolysate.

On the 1st day of storage, the highest lactic acid content was found in LP and LP1.5H (milk with 1.5% collagen protein hydrolysate) milk fermented by *L. paracasei*. Furthermore, depending on the type and dose, collagen also influenced the amount of lactic acid production by the tested probiotic strains. Compared to control milk, more lactic acid was produced by *L. casei* (at day 1) in milk with the addition of bovine collagen at both doses (LC1.5W—milk with 1.5% collagen and LC3.0W—milk with 3% collagen) and hydrolysate at only 3.0% (LC3.0H—milk with 3.0% collagen protein hydrolysate). Similarly, more lactic acid was found in milk with bovine collagen at doses of 1.5 and 3.0% fermented by *L. acidophilus* than the control. The addition of hydrolysate (LA1.5H—milk with 1.5% collagen protein hydrolysate and LA3.0H—milk with 3% collagen protein hydrolysate) resulted in lower lactic acid content on day 1 of cold storage.

The analysis of the lactic acid contents of milk fermented by *L. paracasei* and *L. rhamnosus* showed that, compared with the controls, the collagen was reduced in content.. A tendency was observed that adding the hydrolysate primarily resulted in a reduction of lactic acid concentration but only on day 1 of storage. In contrast, the opposite was observed for milk fermented by *L. acidophilus* LA1.5H and LA3.0H and *L. rhamnosus* LR1.5H (milk with 1.5% collagen protein hydrolysate), where the lactic acid concentration was also lower after 21 days. The ANOVA (Table 5) showed that the lactic acid contents of sheep’s milk depended on storage time, probiotic type, collagen type and the interaction of these factors.

Table 1, Table 2, Table 3 and Table 4 show the results that determined the color parameters of probiotic-fermented sheep’s milk. The analysis of L* lightness results showed that on the 1st and 21st day of storage, the addition of in the form of both bovine and hydrolysate, resulted in a darkening of the milk color. Moreover, increasing storage time also resulted in an increasingly darker milk color. All probiotic sheep’s milk samples were characterized by the proportion of green (−a*) and yellow (+b*) color. The three-factor analysis of variance performed indicated that color parameters were influenced by storage time, bacterial strains, collagen type, and interactions between bacterial strain and storage time as well as interactions between bacterial strain and collagen type (Table 5).

An essential characteristic of lactic acid gel is its ability to retain and bind water. In our study, the addition of bovine collagen only in milk fermented by *L. casei* (LC1.5W and LC3.0W) and *L. acidophilus* (LA1.5W, milk with 1.5% collagen, and LA3.0W, milk with 3.0% collagen) was found to increase syneresis by approximately 1–5% at for both storage durations. This was in contrast with milk fermented by *L. paracasei* and *L. rhamnosus*. However, the addition of hydrolysate was effective in reducing syneresis in each milk compared to its control equivalent. A trend was also observed that syneresis decreased with an increasing dose of hydrolysate.

The hardest gel on the first and twenty-first day of storage corresponded to LP control milk fermented by *L. paracasei*. The high gel hardness of this milk was related to low pH value and increased lactic acid content. Analysis of the milk gel hardness results in Table 2, Table 3 and Table 4 showed a decrease in hardness in the milk samples with bovine collagen and hydrolysate compared to the control samples. The reversed dependence was found only in milk fermented by *L. casei* (Table 1). Analysis of variance confirmed that the texture components (hardness, cohesiveness, springiness) were mainly affected by the type of probiotic strain used, the type of collagen and the interaction of these two factors (Table 5).

### 2.2. Microbiological Analysis

The bacterial count evaluation in control milk showed that the optimum growth conditions in control sheep’s milk were for *L. paracasei* (LP) and *L. rhamnosus* (LR) (Table 6). In both samples of control milk, LP and LR were found to be over 11 log cfu g^−1^ on day 1 of storage. The other two strains (*L. acidophilus* and *L. casei*) were characterized by a weaker growth, obtaining a bacterial cell count of about 9 log cfu g^−1^ on day 1 of storage. Moreover, the growth of *L. casei* and *L. acidophilus* cells on day 1 of storage was not significantly affected by the type and dose of collagen. However, the growth of *L. rhamnosus* and *L. paracasei* strains was influenced mainly by collagen type and dose. LP1.5W, LP3.0W and LP3.0H milk, as well as LR1.5W, LR1.5H and LR3.0H milk, had lower bacterial cell counts on day 1 of storage than controls. The intensive growth of bacterial cells in sheep’s milk in control milk fermented by *L. paracasei* and *L. rhamnosus* was unfortunately not associated with similarly good survival of these bacteria during 21 days of refrigerated storage. After 21 days of storage, a 1.7 to 2.3 log cfu g^−1^ reduction in the cells of these bacteria was found.

Furthermore, the milk with collagen fermented by *L. rhamnosus* and *L. paracasei* also showed a reduction in the bacterial population to about 9 log cfu g^−1^. Only in the LP3.0H milk did the bacterial count remain constant during storage. In contrast, sheep’s milk fermented with *L. acidophilus* and *L. casei* showed good survival during storage, and there was no effect of collagen type and dose on the growth and survival of these strains. Although *L. casei* had the lowest growth in sheep’s milk and low bacterial cell count on day 1 of storage, it showed notable ability to grow under refrigerated conditions during 21 days of storage. An increase in the *L. casei* population (by about 1 log cfu g^−1^) during storage was observed in LC control milk and milk with hydrolysate (LC1.5H and LC3.0H). However, in sheep’s milk with collagen LC1.5W and LC3.0W, the number of bacterial cells did not change significantly during 21 days of storage.

### 2.3. Organoleptic Evaluation

**Figure 1:**
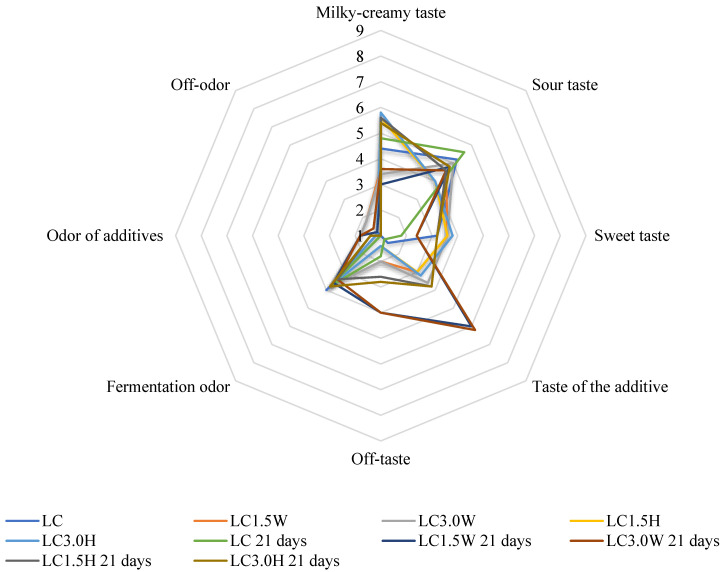
Effect of collagen addition on organoleptic parameters of milk fermented by *L. casei* after 1 and 21 days of cold storage. LC—control milk with *Lacticaseibacillus casei*; LC1.5W—milk with 1.5% collagen and *Lacticaseibacillus casei*; LC3.0W—milk with 3.0% collagen and *Lacticaseibacillus casei*; LC1.5H—milk with 1.5% collagen hydrolysate and *Lacticaseibacillus casei*; LC3.0H—milk with 3.0% collagen hydrolysate and *Lacticaseibacillus casei*.

**Figure 2:**
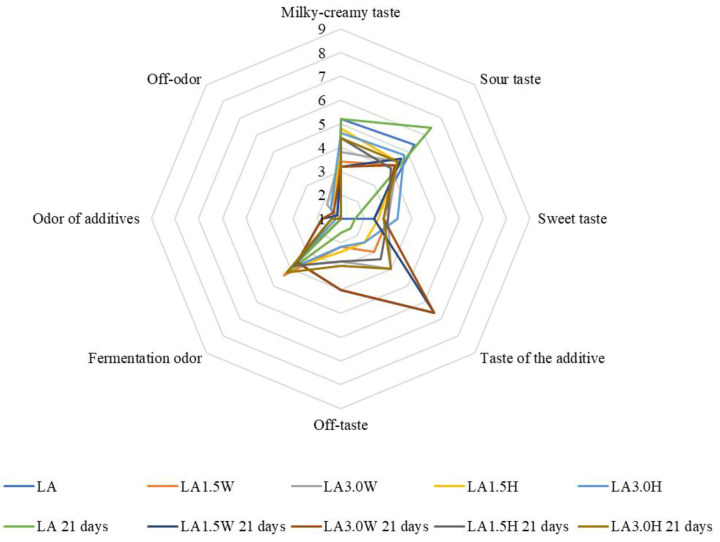
Effect of collagen addition on the organoleptic parameters of milk fermented by *L. acidophilus* after 1 and 21 days of cold storage. LA—control milk with *Lactobacillus acidophilus*; LA1.5W—milk with 1.5% collagen and *Lactobacillus acidophilus*; LA3.0W—milk with 3.0% collagen and *Lactobacillus acidophilus*; LA1.5H—milk with 1.5% collagen hydrolysate and *Lactobacillus acidophilus*; LA3.0H—milk with 3.0% collagen hydrolysate and *Lactobacillus acidophilus*.

**Figure 3:**
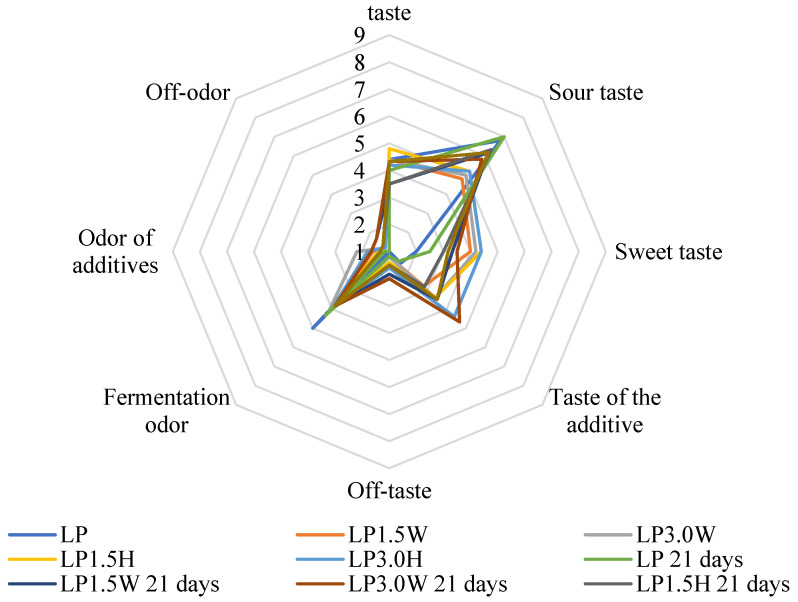
Effect of collagen addition on the organoleptic parameters of milk fermented by *L. paracasei* after 1 and 21 days of cold storage. LP—control milk with *Lacticaseibacillus paracasei;* LP1.5W—milk with 1.5% collagen and *Lacticaseibacillus paracasei;* LP3.0W—milk with 3.0% collagen and *Lacticaseibacillus paracasei*; LP1.5H—milk with 1.5% collagen hydrolysate and *Lacticaseibacillus paracasei*; LP3.0H—milk with 3.0% collagen hydrolysate and *Lacticaseibacillus paracasei*.

**Figure 4:**
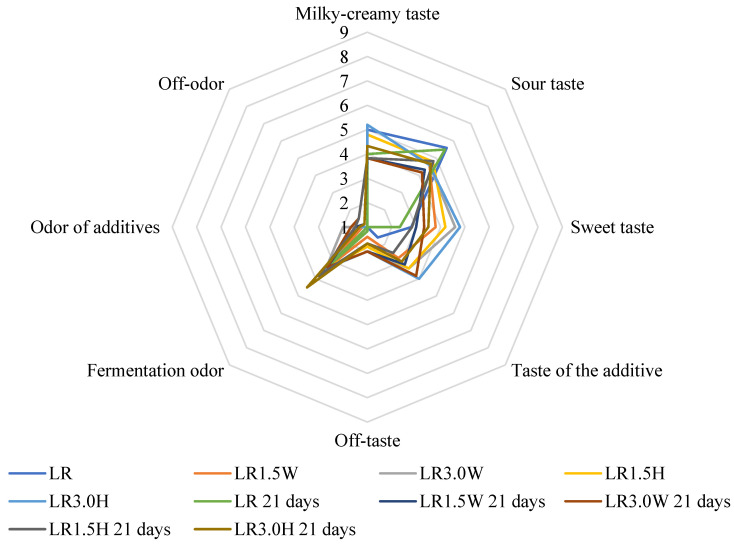
Effect of collagen addition on the organoleptic parameters of milk fermented by *L. rhamnosus* after 1 and 21 days of cold storage. LR—control milk with *Lacticaseibacillus rhamnosus*; LR1.5W—milk with 1.5% collagen and *Lacticaseibacillus rhamnosus*; LR3.0W—milk with 3.0% collagen and *Lacticaseibacillus rhamnosus*; LR1.5H—milk with 1.5% collagen hydrolysate and *Lacticaseibacillus rhamnosus*; LR3.0H—milk with 3.0% collagen hydrolysate and *Lacticaseibacillus rhamnosus*.

The results of the organoleptic evaluation are shown in Figure 1, Figure 2, Figure 3 and Figure 4. The most intense milky–creamy taste at 1 and 21 days of storage corresponded to the milk samples with hydrolysate fermented by *L. casei*. These studies showed that the addition of bovine collagen and hydrolysate increased sweet taste intensity compared to their control counterparts. The sourest taste was found in the control LP milk fermented by *L. paracasei*, and it became yet sourer with prolonged storage. Furthermore, all milk samples with collagen (bovine, hydrolysate) showed an off taste, which was more intense in milk with bovine collagen than hydrolysate, especially after 21 days of storage. Moreover, milk samples with bovine collagen were also characterized by a more intense additive odor and off-odor than those with hydrolysate. The analysis of variance indicated that the main factor influencing all organoleptic characteristics of milk was the type of collagen (Table 6). However, the storage time significantly affected the taste of fermented milk.

## 3. Discussion

Milk contains essential nutrients and provides a good medium for the growth of *Lactobacillus* and *Lacticaseibacillus*. An additional type of amino acids in the form of collagen could stimulate the growth of these probiotic bacteria. Therefore, these probiotic bacteria can be used as starter cultures [35]. Probiotic bacteria grow and maintain viability until substrate (carbohydrates, amino acids, and other nutrients) are depleted; toxic and inhibitory substances (e.g., hydrogen peroxide) accumulate; and the pH value changes to an extent that bacterial growth is affected. Two of the factors affecting the growth and survival of lactic acid bacteria are pH value and temperature [36]. The optimal temperature and pH conditions for the growth of *Lacticaseibacillus* and *Lactobacillus* are 30–40 °C and pH 5.5–6.2, respectively. Some strains show the ability to grow at temperatures ranging from 2 to 53 °C and pH ranging from 4.5 to 7.0 and even below 4.4 (examples are *L. acidophilus* and *L. casei*) [37,38]. In contrast, magnesium leakage from *L. casei* cells occurs at pH < 3.0 [36]. For the growth of *L. rhamnosus*, the optimal pH value is between 6.4 and 6.9, and the minimum pH value is between 4.4 and 3.4, depending on the buffering capacity of the medium [15]. *L. paracasei* (as reported by the manufacturer) survives perfectly in a highly acidic environment (pH = 2.5) [39]. In the studied sheep’s milk, the lowest pH, 3.85, was determined after 21 days of storage in LP milk fermented by *L. paracasei*. In the other samples fermented by other probiotics, the pH value was higher than 4.0, suggesting that this parameter was within the acceptable range.

The viability of probiotic bacteria is one of the most critical factors determining the quality of dairy probiotic products. The acceptable survival rate of probiotic bacteria in our study could be attributed to the probiotic strains’ characteristics. This study showed the best survival rate of *L. casei* in milk with collagen protein hydrolysate. In contrast to our research, Yerlikaya et al. [40] in fermented milk showed the lowest survival of *L. casei* during 30 days of refrigerated storage. A reduction in the bacterial population was found in milk fermented by *L. rhamnosus* and *L. paracasei* (Table 5). A similar effect of collagen hydrolysate on the survival of *L. rhamnosus* was observed in a study by Znamirowska et al. [30]. Da Mata Rigoto et al. [41] showed that adding collagen hydrolysate in probiotic beverages had a minor or no effect on *L. acidophilus* survival, which is also confirmed by our study. In the sheep’s milk yogurts fermented with *L. acidophilus* manufactured by Vianna et al. [42], the pH value on day 1 of storage was 4.67, and on day 21, pH = 4.52. These values are higher than those in Table 2 for LA milk and are comparable to milk with hydrolysate.

In contrast, the addition of 1.5% bovine collagen resulted in a reduction of pH value by 0.14, and the addition of 3.0% collagen resulted in a reduction of pH value by 0.23. Earlier research [43] indicated a slow reduction in pH in milk containing 3.06% and 5.1% collagen, indicating the phenomenon of delayed fermentation caused by collagen addition. Similar results were obtained in milk with different types of collagen, i.e., fish and pork collagen [43].

In our study, the pH value of fermented milk during storage decreased significantly in all analyzed milk samples. Another study [30] showed that fermented cow’s milk with added collagen hydrolysate had a higher pH value than control milk at both 1 and 21 days of storage. In a study by Shori et al. [44], the addition of fish collagen increased the initial titratable acidity (TA%) by approximately 0.2% lactic acid equivalent, which is the most organic acid present in fermented dairy products [44,45]. However, a study by Kavaz and Bakirci [46] reported that the amount of lactic acid in probiotic yogurts increased with storage time, which is also confirmed by the results in Table 1, Table 2, Table 3 and Table 4. During storage, β-galactosidase, which is still active at 0–5 °C, might also be responsible for lowering the pH value. In this case, the pH could decrease below 4.2 [47] which is similar to the trend observed in the current report (Table 3).

Syneresis is a natural separation of liquid and gel which occurs in fermented dairy products such as yogurt, sour milk and kefir [48]. The quality of fermented milk can be controlled by using various texture-enhancing stabilizers. Gelatin and other proteins of animal origin have the ability to impart increased firmness–viscosity to fermented milk by preventing the separation of whey from the acid gel of milk [30,48]. The addition of collagen hydrolysate to cow’s milk was shown to reduce syneresis at 1 and 21 days of storage [30]. In a study by Gerhardt et al. [49], the addition of collagen hydrolysate above 1.0% reduced the rate of syneresis of the milk beverage, improving its stability. In our study, adding collagen protein hydrolysate reduced the syneresis of fermented milk. According to Tribst et al. [50], the gel network formed in sheep’s milk yogurt is characterized by high water retention capacity; moreover, at low temperatures, the action of proteases is reduced, causing the protein degradation to slow down. This probably contributes to the occurrence of less syneresis [50]. Also, storage time significantly affects the amount of whey leakage, as confirmed by Panesar and Shinde [47] and Khorshidi et al. [51] in their studies.

The texture properties of fermented milk may be related to moisture content and protein level [40]. Several studies have reported that the addition of 3.0% collagen protein hydrolysate to cow’s milk increases hardness and adhesion [30]. However, in our study, the addition of hydrolysate and bovine collagen increased hardness only in milk fermented by *L. casei*. This result is crucial for the application of probiotics in the field of fermented milk. Applying different probiotics can provide different hardness and consistency of fermented milk, depending on whether it is designed for drinking or eating with a spoon. The high gel hardness of LC1.5H and LC3.0H milk was most probably due to better cross-linking of the gel bonds or more bacterial exopolysaccharides, which also resulted in very low syneresis and high springiness. Zhang et al. [52] found that *L. casei* has double clusters of EPS (exopolysaccharides), implying a genetic basis for EPS production. Many studies indicate that the increase in EPS–EPS interaction allows the formation of long EPS strands and gives the yogurt appearance and texture, resulting in high probiotic milk hardness [53,54,55]. Probiotic bacteria can impart different textures to the acid gel of fermented milk, due to differences in the amount of organic acids produced [56]. In our study, the calculated Pearson correlation coefficients indicate a significant correlation between pH value and lactic acid content and gel hardness.

Yerlikaya et al. [40] observed changes in viscosity of the resulting milk depending on the probiotic strains used. Milk fermented by *L. acidophilus* had a higher viscosity than milk fermented by *L. casei* [40]. The texture profile of fermented milk is also affected by the type of raw material used, as confirmed in a study by Vianna et al. [42]. Yogurts made from sheep’s milk were characterized by higher firmness and viscosity than those made from cow’s milk [42]. This is due to the higher content of dry matter, proteins and lipids in sheep’s milk, which promotes better matrix network stabilization [42,57]. The protein network of casein micelles retains fat globules and serum during fermentation [50].

Color is an essential attribute of food, and it is the first attribute perceived by consumers serving as a factor in consumer choice. In a study by Mani-López et al. [58], the color parameters L*, a* and b* of yogurt and fermented milk obtained using different probiotic cultures did not change significantly during 35 days of storage. Sheep’s milk fermented by *L. acidophilus* and *L. rhamnosus* obtained by Szajnar et al. [15] corresponded to light color and the proportion of green (−a*) and yellow (+b*) color. However, our study showed that both bovine and hydrolysate collagen caused the darkening of milk color.

One of the criteria for selecting bacterial strains for milk fermentation is the absence of negative effects of their metabolites on sensory quality [59]. According to Szajnar et al. [15], the presence of sweet and sour tastes in the fermented milk was significantly correlated with the type of probiotic bacteria carrying out the fermentation. In a study by Mani-López [58], probiotic yogurts and fermented milk containing *L. casei* were better accepted by panelists than other yogurts. In our research, milk samples with *L. casei* had the most intense milky–creamy taste. It should be noted that increasing the additive dose from 1.5% to 3.0% did not significantly effect the taste and odor of fermented sheep’s milk. In a study by Mani-López [58], probiotic yogurts and fermented milk containing *L. casei* were more acceptable to panelists than other yogurts. According to Sun et al. [60], *L. casei* can significantly contribute to the quality of fermented milk during fermentation, affecting the fermentation process and the formation of metabolites during storage. Acetaldehyde is an important flavor compound that gives fermented milk the odor of green apple [61]. Moreover, 2,3-butanedione and acetoin are important flavor sources in fermented milk. 2,3-butanedione can give fermented milk a unique buttery aroma [62]. Acetoin is converted from 2,3-butanedione by the enzyme diacetyl reductase, giving fermented milk a sweet, cultured and buttery taste [61,63]. According to Gao et al. [64] milk fermented by *L. casei* has a higher content of acetic acid, lactic acid, butyric acid, caproic acid, acetoin, 2-butanone and 2-ethyl-1-hexanol, which may result in higher product acceptance.

A study by Znamirowska et al. [30] reported that control samples of fermented milk using *Bifidobacterium* Bb-12 and *L. rhamnosus* with collagen protein hydrolysate did not differ significantly in organoleptic evaluation parameters at both 1 and 21 days of storage [30]. Similar results were obtained from an organoleptic evaluation of probiotic milk drinks by Da Mata Rigoto et al. [41], demonstrating that the addition of collagen hydrolysate does not significantly modify characteristics such as appearance, aroma, taste or texture. However, in our study, adding collagen increased the intensity of sweetness and off-taste, which might be related to the animal species by which the collagen is produced.

Organoleptic characteristics, such as perceptibility of sweetness and off-taste, are influenced by the type of raw material used (milk), the type of probiotic strains used for fermentation and the type of additives used (collagen). In a study by Znamirowska et al. [30] on probiotic milk fermented by *L. rhamnosus* with bovine collagen hydrolysate, the panellists reported a slightly sweet taste at 1 and 21 days of storage. Moreover, according to Soomro et al. [65], applying stabilizers like gelatin to milk preferably increases the creamy taste. In a study by Shori et al. [44], the addition of fish collagen reduced aroma intensity in yogurt. However, Karim and Bhat [66] found that fish collagen had a beneficial effect in improving the organoleptic properties of yogurt, such as taste and texture. Similarly, Shori et al. [67] found that fresh and 7-day-old yogurt with fish collagen had higher overall acceptability than yogurt without fish collagen.

In our study, the applied bovine collagen and hydrolysate differed in amino acid composition. The hydrolysate had a higher glycine content (24.70 g/100 g) than bovine collagen (20.60 g/100 g). Glycine was found to be as sweet as glucose [68]. A higher glycine content increased the intensity of the sweet taste. Moreover, Razak et al. [69] and Chen and Zhang [70] state that the taste of glycine is sweet like glucose due to its sweet nature. Three proteinogenic amino acids have been reported to elicit a sweet taste in humans, including l-glycine, l-alanine, and l-threonine. The TAS1R2/TAS1R3 receptor is able to detect a wide chemical variety of sweet-tasting compounds, including carbohydrates (such as fructose, glucose and sucrose) and natural (stevioside) and artificial (such as aspartame, saccharin and cyclamate) sweeteners. The TAS1R2/TAS1R3 receptor is also activated by the sweet amino acids in both the l- and d-configurations mentioned above, such as glycine, l-alanine and d-tryptophan [71].

## 4. Materials and Methods

### 4.1. Materials

The material for the production of probiotic fermented milk was raw sheep’s milk collected in May 2021 from farms in the Zakopane and Nowy Sącz region (Poland), with the following chemical composition: fat, 7.31% ± 0.36%; protein, 6.20% ± 0.21%; lactose, 4.59% ± 0.09%; total solids, 19.53% ± 0.44; pH, 6.30 ± 0.43; freezing point, −0.678 °C ± 0.015 °C; density, 1.036 g/l ± 0.002 g/l; color components—L*: 92.17 ± 1.93, a*: −2.45 ± 0.37, b*: 9.13 ± 0.63, C: 9.46 ± 0.69, h°: 105.13 ± 1.29; total bacterial count (TBC), 710,166.60 ± 16,674.13 cfu in 1 mL; somatic cell count (SCC), 339,833.33 ± 26,753.82 in 1 mL. The methods of milk analysis are presented in Section 4.3.1.

Two types of collagens were used as additives: 100% collagen protein hydrolysate (Vitagel-Collagen, Superior, Dobre Miasto, Poland) and bovine collagen—100% natural (FH Kol-Pol, Dębica, Poland). Four strains of probiotic bacteria were used for milk fermentation: *Lacticaseibacillus casei* 431^®^ (Chr. Hansen, Hoersholm, Denmark), *Lactobacillus acidophilus* LA-5^®^ (Chr. Hansen, Hoersholm, Denmark), *Lacticaseibacillus paracasei* Lpc-37^®^ (Danisco, DuPont, Copenhagen, Denmark) and *Lacticaseibacillus rhamnosus* Lr-32^®^ (Danisco, DuPont, Copenhagen, Denmark).

IBCm Bacto Kit 500 and IBCm SCC Kit reagents for the determination of TBC and SCC were purchased from Bentley Instruments Inc. (Chaska, MN, USA). MRS agars and peptone water came from Biocorp (Warszawa, Poland). Sodium hydroxide and phenolphthalein were purchased from Chempur (Piekary Śląskie, Poland).

All of the reagents used were of analytical reagent grade.

### 4.2. Fermented Milk Production

Raw sheep’s milk was pasteurized at 85 °C for 30 min and divided into 20 groups due to the added probiotic (*Lacticaseibacillus casei, Lactobacillus acidophilus, Lacticaseibacillus paracasei, Lacticaseibacillus rhamnosus*) and different dosages (1.5% and 3.0%) and types of collagen (hydrolysate and bovine collagen). The starter of bacterial cultures was prepared according to the method described by Szajnar et al. [15]. Five groups of milk were inoculated with one probiotic, in which the first group was without collagen (control), the second group contained the addition of 1.5% collagen hydrolysate, the third group had 3.0% collagen hydrolysate added, the fourth group had 1.5% bovine collagen added and the fifth group had 3.0% bovine collagen added. The milk–collagen mixture was then heated to 60 °C, homogenized at 20 MPa (Nuoni GJJ-0.06/40, Shanghai, China) and re-pasteurized according to the method of Ramasubramanian et al. [72] (EC) and Commission Regulation (EC) No. 1662/2006 [73] with modifications (at 85 °C for 10 min). After heat treatment, the milk samples were cooled to inoculation temperature (37 ± 1 °C). A total of 20 batches of milk were obtained, according to Table 7.

Each sample was mixed thoroughly, poured into 100 mL plastic containers and fermented in an incubator at 37 ± 1 °C in order to obtain a pH value of 4.6 ± 0.1 (12–15 h). After this period, the fermented milk was cooled to 5 °C (ILW 115 Refrigerated Incubator, POL-EKO Aparatura, Wodzisław Śląski, Poland) and evaluated on days 1 and 21 of cold storage. The experiment was repeated three times, and all analyses were performed in five replicates each time.

### 4.3. Methods of Analyses

#### 4.3.1. Milk Analysis

Chemical composition and freezing point determinations were performed using a Bentley B-150 milk and milk product analyzer (Bentley Instruments Inc., Chaska, MN, USA), and total bacterial count (TBC) and somatic cell count (SCC) were determined using a semi-automated Bacto Count IBC M/SCC (Bentley Instruments Inc., Chaska, MN, USA). The density of sheep’s milk was performed at a temperature of 20 °C, according to the method used by Raţu et al. [74]. The pH value was determined using a Toledo FiveEasy digital pH meter (Mettler Toledo, Greifensee, Switzerland) using an InLab^®^Solids Pro-ISM electrode (Mettler Toledo, Greifensee, Switzerland). The color of milk was determined by a colorimeter (Precision Colorimeter, Model NR 145, Shenzhen, China) using the CIE L*a*b* system (as described in Evaluation of Color).

#### 4.3.2. Physicochemical Properties of Fermented Milk

##### Acidity and pH Measurement

The pH value in milk after adding collagen and collagen hydrolysate and after fermentation was determined by pH-meter FiveEasy (Mettler Toledo, Greifensee, Switzerland) using InLab^®^Solids Pro-ISM electrode (Mettler Toledo, Greifensee, Switzerland) [75] Lactic acid content was determined according to the method described by Jemaa et al. [76]. Fermented milk samples were titrated with 0.1 M NaOH in the presence of phenolphthalein as an indicator. Lactic acid content was expressed as g lactic acid L^−1^.

##### Evaluation of Color

The color was determined by a colorimeter (Precision Colorimeter, Model NR 145, Shenzhen, China) using the CIE L*a*b* system. The following parameters were determined: L*—as lightness (from 0—black to 100—white), a*—as color from red (+) to green (−), b*—as color from yellow (+) to blue (−), C—as color purity and intensity and h^0^—as color hue [24]. Before measurement, the device was calibrated on a white reference standard [77].

##### Syneresis

Syneresis was determined by the centrifuge method using the laboratory refrigeration centrifuge LMC-4200R (Biosan SIA, Riga, Latvia) according to Santillan-Urquiza et al. [78] method with modifications: 10 g of product was transferred into a 50 mL plastic tube and centrifuged at 4000 rpm for 10 min. The separated whey was weighed and converted to percentages.

##### Texture Profile

Texture profile analysis was determined by an instrumental method using CT3 Texture Analyzer with Texture Pro CT software (Brookfield AMETEK, Berwyn, PA, USA), according to Znamirowska et al. [79]. The test was conducted on a 100 mL sample of fermented milk. The cylindrical dimensions of the sample were as follows: 66 mm × 33.86 mm. The sample temperature was 8 °C. Settings used: trigger load 0.1 N, test speed 1 mm/s, TABTKIT table, probe TA3/100 (acrylic cylinder—diameter 35 mm); test termination distance: 15 mm. Parameters measured: hardness [N], cohesiveness, springiness [mm].

#### 4.3.3. Microbiological Analysis

The probiotic bacteria load of *Lacticaseibacillus casei* 431, *Lactobacillus acidophilus* LA-5, *Lacticaseibacillus paracasei* Lpc-37 and *Lacticaseibacillus rhamnosus* Lr-32 was determined by plate method using MRS agar according to the method of Znamirowska et al. [26] and Lima et al. [77]. Incubation was conducted under anaerobic conditions at 37 °C for 72 h in a vacuum desiccator and GENbox anaerator (Biomerieux, Warsaw, Poland). After incubation, colonies were counted using a colony counter (TYP J-3, Chemland, Stagard Szczeciński, Poland). The number of viable bacterial cells was expressed as log cfu g^−1^.

#### 4.3.4. Organoleptic Evaluation

The organoleptic evaluation was carried out by a trained panel for probiotic fermented milk enriched with collagen and collagen hydrolysate at 1 and 21 days of refrigerated storage. Parameters were evaluated on a 9-point scale (from 1 = undetectable to 9 = very intense). The following parameters were evaluated: texture, color, smoothness, presence of milky–creamy taste, sour taste, sweet taste, additives taste and off-taste (bitter, metallic), and the presence of sour odor, additives odor and off-odor [1,80].

Definition of the attributes in descriptive organoleptic analysis of fermented milk [81]:

Milky–creamy taste: the taste stimulated by milk powder

Sour taste: the taste stimulated by lactic acid

Taste of additives: the taste stimulated by added collagen depending on the collagen type

Sweet taste: the taste stimulated by sucrose

Off-taste: the occurrence of an atypical taste similar to meat broth

Fermentation odor: the intensity of odor associated with sour milk, i.e., lactic acid

Odor of additives: odor characteristic stimulated by added collagen depending on the collagen type

Off-odor: the occurrence of an atypical odor similar to meat broth

### 4.4. Statistical Analysis

From the obtained results, the mean, standard deviation and Pearson’s correlation coefficient were calculated using Statistica v. 13.1 software (StatSoft, Tulsa, Oklahoma, USA). One-way, two-way and three-way ANOVA was conducted to investigate the overall effect of collagen type and dose, storage time (days) and type of bacteria on the properties of probiotic fermented sheep’s milk. The significance of the differences between means was estimated by the Tukey test (*p* < 0.05).

## 5. Conclusions

This study confirmed the possibility of using bovine collagen and collagen hydrolysate in the production of probiotic sheep’s milk. The addition of collagen changed the pH value of milk before fermentation. After fermentation, the number of colony-forming probiotic bacterial cells was higher than 8 log cfu g^−1^. In sheep’s milk fermented by *L. acidophilus* and *L. casei,* there was better survival of bacteria during storage, and there was no effect of collagen dose on the growth and survival of both strains. Moreover, it was shown that in milk with hydrolysate, there were beneficial growth conditions for *L. casei* even during refrigerated storage. This is crucial information for the dairy industry, since using collagen hydrolysate and *L. casei* to ferment sheep’s milk provides a sufficient number of probiotic bacteria cells, increasing storage duration. However, adding collagen to sheep’s milk resulted in a reduction in color lightness, a situation which could be easily improved by using chocolate or fruit flavorings. Collagen enhanced the intensity of the sweet taste due to the presence of sweet glycine, thus, the addition of sugar could be reduced. The most intense milky–creamy taste was found in milk with hydrolysate fermented by *L. casei.* However, the addition of collagen (bovine, hydrolysate) caused a slight off-taste and off-odor to the milk, although appropriate flavor additives could successfully mask this.

## Figures and Tables

**Table 1:** Acidity, syneresis, color and texture of sheep’s milk fermented by *Lacticaseibacillus casei*.

Properties		Storage Time (Days)	LC	LC1.5W	LC3.0W	LC1.5H	LC3.0H
pH		1	4.45 ^a^ ± 0.09	4.56 ^a^ ± 0.02	4.54 ^a^ ± 0.01	4.52 ^a^ ± 0.04	4.60 ^b^ ± 0.01
		21	4.11 ^a^ ± 0.00	4.11 ^a^ ± 0.01	4.10 ^a^ ± 0.01	4.11 ^a^ ± 0.01	4.13 ^b^ ± 0.01
Lactic acid, g L^−1^		1	1.21 ^a^ ± 0.01	1.26 ^b^ ± 0.20	1.27 ^b^ ± 0.04	1.20 ^a^ ± 0.01	1.30 ^b^ ± 0.04
		21	1.22 ^a^ ± 0.01	1.56 ^b^ ± 0.02	1.58 ^b^ ± 0.03	1.53 ^b^ ± 0.08	1.58 ^b^ ± 0.01
Syneresis, %		1	25.74 ^b^ ± 0.61	26.45 ^b^ ± 0.80	25.73 ^b^ ± 0.51	9.82 ^a^ ± 1.52	8.00 ^a^ ± 1.23
		21	22.61 ^c^ ± 1.08	25.62 ^d^ ± 0.63	27.59 ^d^ ± 1.64	13.52 ^b^ ± 1.49	5.97 ^a^ ± 1.21
Color	L*	1	93.70 ^a^ ± 1.81	93.19 ^a^ ± 2.03	92.29 ^a^ ± 0.98	93.12 ^a^ ± 1.00	93.26 ^a^ ± 0.77
		21	93.04 ^b^ ± 0.72	92.02 ^b^ ± 0.77	90.90 ^a^ ± 0.50	92.68 ^b^ ± 0.29	92.82 ^b^ ± 0.73
	a*	1	−2.50 ^a^ ± 0.70	−2.13 ^a^ ± 0.07	−1.67 ^b^ ± 0.14	−1.47 ^bc^ ± 0.19	−1.30 ^c^ ± 0.25
		21	−2.53 ^c^ ± 0.14	−2.27 ^c^ ± 0.21	−1.93 ^a^ ± 0.14	−2.07 ^b^ ± 0.09	−2.04 ^b^ ± 0.22
	b*	1	11.12 ^c^ ± 0.37	9.59 ^b^ ± 1.12	8.62 ^a^ ± 0.58	11.51 ^c^ ± 0.35	11.42 ^c^ ± 0.10
		21	11.73 ^b^ ± 0.53	12.29 ^bc^ ± 0.26	12.86 ^c^ ± 0.30	10.44 ^a^ ± 0.15	10.31 ^a^ ± 0.35
	C	1	11.42 ^c^ ± 0.25	9.83 ^b^ ± 1.09	8.78 ^a^ ± 0.59	11.61 ^c^ ± 0.37	11.48 ^c^ ± 0.09
		21	11.99 ^b^ ± 0.49	12.50 ^bc^ ± 0.29	13.01 ^c^ ± 0.28	10.64 ^a^ ± 0.15	10.51 ^a^ ± 0.37
	h^0^	1	102.73 ^b^ ± 3.76	102.66 ^b^ ± 1.30	100.96 ^b^ ± 0.26	97.29 ^a^ ± 0.82	96.61 ^a^ ± 1.34
		21	102.16 ^c^ ± 1.23	100.45 ^b^ ± 0.79	98.55 ^a^ ± 0.83	101.22 ^c^ ± 0.48	101.15 ^c^ ± 1.00
Hardness, N		1	0.58 ^a^ ± 0.10	0.68 ^a^ ± 0.02	0.75 ^b^ ± 0.01	1.28 ^c^ ± 0.05	1.35 ^c^ ± 0.05
		21	1.01 ^a^ ± 0.10	1.13 ^a^ ± 0.19	1.13 ^a^ ± 0.14	1.78 ^b^ ± 0.02	2.05 ^b^ ± 0.18
Cohesiveness		1	0.84 ^b^ ± 0.10	0.87 ^b^ ± 0.02	0.82 ^b^ ± 0.01	0.53 ^a^ ± 0.03	0.51 ^a^ ± 0.03
		21	0.73 ^b^ ± 0.06	0.81 ^b^ ± 0.06	0.76 ^b^ ± 0.02	0.48 ^a^ ± 0.03	0.46 ^a^ ± 0.03
Springiness, mm		1	14.08 ^a^ ± 1.19	14.57 ^a^ ± 0.43	14.53 ^a^ ± 0.09	14.42 ^a^ ± 0.44	15.36 ^a^ ± 0.80
		21	14.14 ^a^ ± 0.94	14.24 ^a^ ± 0.83	14.12 ^a^ ± 0.44	14.69 ^a^ ± 0.44	15.45 ^a^ ± 0.20

Mean ± standard deviation; n = 20; ^a–d^—Mean values denoted in rows by different letters differ significantly at (*p* ≤ 0.05). Storage time: 1—after fermentation, 21—after 21 days; LC—control milk with *Lacticaseibacillus casei*; LC1.5W—milk with 1.5% collagen and *Lacticaseibacillus casei*; LC3.0W—milk with 3.0% collagen and *Lacticaseibacillus casei*; LC1.5H—milk with 1.5% collagen hydrolysate and *Lacticaseibacillus casei*; LC3.0H—milk with 3.0% collagen hydrolysate and *Lacticaseibacillus casei*.

**Table 2:** Acidity, syneresis, color and texture of sheep’s milk fermented by *Lactobacillus acidophilus*.

Properties		Storage Time (Days)	LA	LA1.5W	LA3.0W	LA1.5H	LA3.0 H
pH		1	4.28 ^a^ ± 0.02	4.35 ^b^ ± 0.01	4.44 ^c^ ± 0.00	4.49 ^d^ ± 0.02	4.74 ^e^ ± 0.01
		21	4.23 ^a^ ± 0.01	4.30 ^b^ ± 0.01	4.39 ^c^ ± 0.03	4.52 ^d^ ± 0.01	4.77 ^e^ ± 0.03
Lactic acid, g L^−1^		1	1.29 ^c^ ± 0.00	1.31 ^d^ ± 0.01	1.34 ^e^ ± 0.01	1.23 ^b^ ± 0.01	1.19 ^a^ ± 0.01
		21	1.48 ^d^ ± 0.01	1.37 ^c^ ± 0.03	1.44 ^d^ ± 0.03	1.31 ^b^ ± 0.01	1.22 ^a^ ± 0.01
Syneresis, %		1	30.12 ^c^ ± 0.61	32.21 ^d^ ± 0.39	34.07 ^d^ ± 1.00	27.97 ^b^ ± 0.66	23.33 ^a^ ± 1.31
		21	30.80 ^c^ ± 0.21	31.16 ^c^ ± 0.67	31.85 ^c^ ± 0.53	23.94 ^b^ ± 0.61	21.19 ^a^ ± 0.22
Color	L*	1	94.46 ^c^ ± 0.35	91.69 ^a^ ± 0.45	90.75 ^a^ ± 0.90	92.22 ^b^ ± 0.44	92.48 ^b^ ± 0.65
		21	93.99 ^c^ ± 0.43	90.88 ^a^ ± 0.98	89.51 ^a^ ± 0.95	91.60 ^ab^ ± 1.15	92.01 ^b^ ± 0.33
	a*	1	−2.20 ^a^ ± 0.06	−2.33 ^b^ ± 0.05	−2.13 ^a^ ± 0.06	−2.15 ^a^ ± 0.09	−2.14 ^a^ ± 0.20
		21	−1.94 ^b^ ± 0.07	−1.94 ^b^ ± 0.11	−2.04 ^a^ ± 0.11	−2.07 ^a^ ± 0.18	−1.86 ^c^ ± 0.08
	b*	1	10.61 ^a^ ± 0.06	12.08 ^b^ ± 0.58	12.33 ^c^ ± 0.17	11.59 ^b^ ± 0.26	11.08 ^a^ ± 0.40
		21	11.45 ^a^ ± 0.08	12.04 ^ab^ ± 0.36	13.06 ^b^ ± 0.70	11.90 ^a^ ± 0.45	11.45 ^a^ ± 0.16
	C	1	10.84 ^a^ ± 0.06	12.30 ^c^ ± 0.27	12.51 ^c^ ± 0.16	11.79 ^b^ ± 0.25	11.28 ^b^ ± 0.36
		21	11.62 ^a^ ± 0.08	12.20 ^b^ ± 0.35	13.23 ^c^ ± 0.68	12.06 ^b^ ± 0.42	11.68 ^a^ ± 0.51
	h^0^	1	101.70 ^b^ ± 0.37	100.95 ^b^ ± 0.52	99.79 ^a^ ± 0.36	100.51 ^b^ ± 0.55	100.95 ^b^ ± 1.36
		21	99.63 ^b^ ± 0.39	99.20 ^ab^ ± 0.73	98.88 ^a^ ± 0.67	99.66 ^b^ ± 0.58	99.30 ^ab^ ± 0.49
Hardness, N		1	1.03 ^c^ ± 0.01	0.84 ^b^ ± 0.05	0.84 ^b^ ± 0.03	0.76 ^a^ ± 0.04	0.83 ^b^ ± 0.15
		21	1.31 ^d^ ± 0.04	1.00 ^c^ ± 0.11	0.65 ^a^ ± 0.21	0.88 ^bc^ ± 0.03	0.82 ^b^ ± 0.03
Cohesiveness		1	0.52 ^a^ ± 0.02	0.86 ^c^ ± 0.02	0.94 ^d^ ± 0.04	0.52 ^ab^ ± 0.06	0.48 ^a^ ± 0.04
		21	0.43 ^a^ ± 0.01	0.84 ^c^ ± 0.01	1.01 ^d^ ± 0.06	0.57 ^ab^ ± 0.03	0.47 ^a^ ± 0.07
Springiness, mm		1	14.75 ^b^ ± 0.07	14.75 ^b^ ± 0.69	14.56 ^b^ ± 0.33	13.84 ^ab^ ± 0.20	12.48 ^a^ ± 0.72
		21	12.83 ^a^ ± 0.83	13.79 ^b^ ± 0.34	14.64 ^c^ ± 0.42	13.59 ^ab^ ± 0.19	12.72 ^a^ ± 0.62

Mean ± standard deviation; n = 20; ^a–e^—Mean values denoted in rows by different letters differ significantly at (*p* ≤ 0.05). Storage time: 1—after fermentation, 21—after 21 days; LA—control milk with *Lactobacillus acidophilus*; LA1.5W—milk with 1.5% collagen and *Lactobacillus acidophilus*; LA3.0W—milk with 3.0% collagen and *Lactobacillus acidophilus*; LA1.5H—milk with 1.5% collagen hydrolysate and *Lactobacillus acidophilus*; LA3.0H—milk with 3.0% collagen hydrolysate and *Lactobacillus acidophilus*.

**Table 3:** Acidity, syneresis, color and texture of sheep’s milk fermented by *Lacticaseibacillus paracasei*.

Properties		Storage Time (days)	LP	LP1.5W	LP3.0W	LP1.5H	LP3.0H
pH		1	4.15 ^a^ ± 0.02	4.42 ^b^ ± 0.01	4.54 ^c^ ± 0.01	4.53 ^c^ ± 0.02	4.59 ^d^ ± 0.01
		21	3.85 ^a^ ± 0.01	3.94 ^b^ ± 0.03	4.05 ^c^ ± 0.02	3.99 ^b^ ± 0.04	4.09 ^c^ ± 0.01
Lactic acid, g L^−1^		1	1.40 ^c^ ± 0.01	1.29 ^a^ ± 0.01	1.40 ^c^ ± 0.01	1.28 ^a^ ± 0.01	1.37 ^b^ ± 0.01
		21	1.64 ^a^ ± 0.02	1.68 ^b^ ± 0.02	1.74 ^c^ ± 0.04	1.72 ^c^ ± 0.02	1.68 ^b^ ± 0.02
Syneresis, %		1	17.93 ^d^ ± 0.55	14.79 ^c^ ± 1.17	8.66 ^a^ ± 1.50	12.77 ^b^ ± 1.67	11.91 ^b^ ± 0.43
		21	15.71 ^c^ ± 1.19	15.54 ^c^ ± 1.99	7.57 ^a^ ± 1.46	11.70 ^b^ ± 1.85	11.56 ^b^ ± 1.72
Color	L*	1	94.36 ^b^ ± 0.22	93.71 ^a^ ± 0.34	93.77 ^a^ ± 0.28	92.92 ^a^ ± 0.58	93.26 ^a^ ± 0.23
		21	93.42 ^b^ ± 0.20	92.56 ^a^ ± 0.22	92.84 ^a^ ± 0.39	92.81 ^a^ ± 0.33	92.59 ^a^ ± 0.98
	a*	1	−0.87 ^b^ ± 0.05	−0.81 ^ab^ ± 0.03	−0.82 ^ab^ ± 0.11	−0.75 ^a^ ± 0.05	−0.86 ^b^ ± 0.05
		21	−1.98 ^a^ ± 0.05	−2.01 ^a^ ± 0.12	−1.96 ^a^ ± 0.19	−2.09 ^a^ ± 0.16	−2.06 ^a^ ± 0.12
	b*	1	10.97 ^a^ ± 0.08	11.04 ^a^ ± 0.09	11.06 ^a^ ± 0.03	11.23 ^b^ ± 0.04	11.20 ^b^ ± 0.04
		21	11.71 ^a^ ± 0.17	11.74 ^a^ ± 0.18	11.39 ^a^ ± 0.19	12.04 ^b^ ± 0.33	12.31 ^b^ ± 0.46
	C	1	11.00 ^a^ ± 0.08	11.23 ^ab^ ± 0.17	11.10 ^a^ ± 0.10	11.26 ^b^ ± 0.04	11.24 ^b^ ± 0.03
		21	11.88 ^a^ ± 0.17	11.91 ^a^ ± 0.19	11.56 ^a^ ± 0.21	12.23 ^b^ ± 0.13	12.66 ^b^ ± 0.21
	h^0^	1	94.55 ^b^ ± 0.07	94.94 ^b^ ± 0.24	94.24 ^a^ ± 0.74	93.82 ^a^ ± 0.29	94.23 ^a^ ± 0.03
		21	99.57 ^b^ ± 0.25	99.70 ^b^ ± 0.48	99.84 ^b^ ± 0.57	99.90 ^b^ ± 0.79	99.04 ^a^ ± 0.03
Hardness, N		1	1.52 ^b^ ± 0.14	1.04 ^b^ ± 0.04	0.93 ^a^ ± 0.03	0.87 ^a^ ± 0.05	1.03 ^ab^ ± 0.12
		21	1.87 ^d^ ± 0.03	1.46 ^c^ ± 0.04	1.13 ^b^ ± 0.01	1.07 ^a^ ± 0.03	1.36 ^c^ ± 0.07
Cohesiveness		1	0.47 ^a^ ± 0.02	0.58 ^b^ ± 0.02	0.60 ^b^ ± 0.07	0.58 ^b^ ± 0.01	0.62 ^b^ ± 0.06
		21	0.50 ^a^ ± 0.02	0.49 ^a^ ± 0.05	0.59 ^b^ ± 0.03	0.58 ^b^ ± 0.03	0.50 ^ab^ ± 0.08
Springiness, mm		1	14.62 ^ab^ ± 0.16	14.80 ^b^ ± 0.13	14.38 ^a^ ± 0.31	14.51 ^a^ ± 0.14	15.21 ^b^ ± 0.37
		21	15.03 ^a^ ± 0.17	14.81 ^a^ ± 0.84	15.14 ^a^ ± 0.47	14.41 ^a^ ± 0.56	14.36 ^a^ ± 0.88

Mean ± standard deviation; n = 20; ^a–d^—Mean values denoted in rows by different letters differ significantly at (*p* ≤ 0.05). Storage time: 1—after fermentation, 21—after 21 days; LP—control milk with *Lacticaseibacillus paracasei;* LP1.5W—milk with 1.5% collagen and *Lacticaseibacillus paracasei;* LP3.0W—milk with 3.0% collagen and *Lacticaseibacillus paracasei*; LP1.5H—milk with 1.5% collagen hydrolysate and *Lacticaseibacillus paracasei*; LP3.0H—milk with 3.0% collagen hydrolysate and *Lacticaseibacillus paracasei*.

**Table 4:** Acidity, syneresis, color and texture of sheep’s milk fermented by *Lacticaseibacillus rhamnosus*.

Properties		Storage Time (Days)	LR	LR1.5W	LR3.0W	LR1.5H	LR3.0H
pH		1	4.27 ^a^ ± 0.03	4.52 ^b^ ± 0.02	4.58 ^c^ ± 0.04	4.59 ^c^ ± 0.04	4.64 ^c^ ± 0.02
		21	4.11 ^a^ ± 0.05	4.16 ^a^ ± 0.03	4.40 ^c^ ± 0.06	4.35 ^bc^ ± 0.05	4.31 ^b^ ± 0.02
Lactic acid, g L^−1^		1	1.35 ^c^ ± 0.02	1.27 ^b^ ± 0.01	1.30 ^bc^ ± 0.02	1.22 ^a^ ± 0.05	1.31 ^c^ ± 0.02
		21	1.46 ^b^ ± 0.04	1.45 ^b^ ± 0.07	1.31 ^a^ ± 0.02	1.30 ^a^ ± 0.05	1.42 ^b^ ± 0.01
Syneresis, %		1	29.80 ^d^ ± 0.35	26.17 ^c^ ± 0.72	13.02 ^a^ ± 0.41	16.46 ^b^ ± 1.23	12.38 ^a^ ± 1.36
		21	21.45 ^d^ ± 0.27	18.46 ^c^ ± 0.70	16.14 ^b^ ± 0.43	16.74 ^b^ ± 1.01	5.19 ^a^ ± 1.13
Color	L*	1	93.43 ^b^ ± 0.84	91.11 ^a^ ± 0.86	91.31 ^a^ ± 0.67	90.54 ^a^ ± 4.84	92.58 ^b^ ± 0.48
		21	92.88 ^b^ ± 0.28	91.10 ^a^ ± 1.07	90.99 ^a^ ± 0.34	90.38 ^a^ ± 0.44	92.18 ^a^ ± 0.92
	a*	1	−0.45 ^b^ ± 0.24	−0.38 ^b^ ± 0.50	−0.68 ^b^ ± 0.13	−0.90 ^a^ ± 0.34	−1.02 ^a^ ± 0.44
		21	−2.00 ^a^ ± 0.13	−2.00 ^a^ ± 0.15	−1.98 ^a^ ± 0.16	−1.89 ^a^ ± 0.21	−2.08 ^a^ ± 0.15
	b*	1	10.75 ^a^ ± 0.53	11.54 ^ab^ ± 0.47	12.17 ^b^ ± 0.24	12.08 ^b^ ± 0.21	12.05 ^b^ ± 0.82
		21	11.97 ^b^ ± 0.15	12.32 ^b^ ± 0.37	12.20 ^b^ ± 0.22	12.09 ^b^ ± 0.31	12.21 ^a^ ± 0.25
	C	1	10.77 ^a^ ± 0.52	11.55 ^ab^ ± 0.48	12.19 ^b^ ± 0.24	12.12 ^b^ ± 0.21	12.11 ^b^± 0.79
		21	12.14 ^a^ ± 0.14	12.19 ^a^ ± 0.35	12.26 ^a^ ± 0.23	12.13 ^a^ ± 0.31	12.19 ^a^ ± 0.23
	h^0^	1	92.43 ^b^ ± 1.36	88.52 ^a^ ± 1.72	86.79 ^a^ ± 0.63	85.70 ^a^ ± 1.50	94.98 ^b^ ± 2.35
		21	99.42 ^a^ ± 0.63	99.22 ^a^ ± 0.87	99.30 ^a^ ± 0.65	98.97 ^a^ ± 1.03	99.74 ^b^ ± 0.97
Hardness, N		1	1.40 ^c^ ± 0.02	0.94 ^b^ ± 0.04	0.85 ^a^ ± 0.01	0.93 ^b^ ± 0.01	1.40 ^c^ ± 0.06
		21	1.54 ^d^ ± 0.08	1.19 ^c^ ± 0.01	1.01 ^a^ ± 0.01	1.11 ^b^ ± 0.02	1.82 ^e^ ± 0.06
Cohesiveness		1	0.47 ^a^ ± 0.01	0.59 ^b^ ± 0.02	0.68 ^c^ ± 0.07	0.59 ^b^ ± 0.08	0.50 ^a^ ± 0.03
		21	0.44 ^a^ ± 0.06	0.59 ^b^ ± 0.03	0.66 ^c^ ± 0.07	0.64 ^c^ ± 0.02	0.49 ^a^ ± 0.05
Springiness, mm		1	13.90 ^a^ ± 0.10	14.20 ^a^ ± 0.12	13.80 ^a^ ± 0.41	14.35 ^a^ ± 0.54	14.34 ^a^ ± 0.53
		21	13.99 ^a^ ± 0.49	14.20 ^a^ ± 0.58	14.16 ^a^ ± 0.52	14.63 ^a^ ± 0.23	14.13 ^a^ ± 0.37

Mean ± standard deviation; n = 20; ^a–e—^Mean values denoted in rows by different letters differ significantly at (*p* ≤ 0.05). Storage time: 1—after fermentation, 21—after 21 days; LR—control milk with *Lacticaseibacillus rhamnosus*; LR1.5W—milk with 1.5% collagen and *Lacticaseibacillus rhamnosus*; LR3.0W—milk with 3.0% collagen and *Lacticaseibacillus rhamnosus*; LR1.5H—milk with 1.5% collagen hydrolysate and *Lacticaseibacillus rhamnosus*; LR3.0H—milk with 3.0% collagen hydrolysate and *Lacticaseibacillus rhamnosus*.

**Table 5:** Analysis of variance (ANOVA) *p*-values on the effects of probiotic strains, storage time and type of collagen on pH, total acidity, syneresis, color (L*, a*, b*, C, h^0^) hardness, cohesiveness, springiness, bacterial cell count, milky–creamy taste, sour taste, sweet taste, taste of the additive, off-taste, fermentation odor and off-odor of fermented sheep’s milk.

	Probiotic Strains; *p*-Values	Storage Time; *p*-Values	Type of Collagen; *p*-Values	Probiotic Strains * Storage Time; *p*-Values	Probiotic Strains * Type of Collagen; *p*-Values	Storage Time * Type of Collagen; *p*-Values	Probiotic Strains * Storage Time * Type of Collagen; *p*-Values
pH	↑0.0000	↑0.0000	↑0.0000	↑0.0000	↑0.0000	↑0.0421	↑0.0230
Lactic acid	↑0.0052	↑0.0037	↑0.0039	↑0.0007	↑0.0012	↑0.0014	↑0.0030
Syneresis	↑0.0000	n.s.0.1558	↑0.0000	↑0.0026	↑0.0000	↑0.0472	↑0.0039
L*	↑0.0011	↑0.0095	↑0.0000	↑0.0000	↑0.0000	n.s.0.8062	n.s.0.2964
a*	↑0.0000	↑0.0000	↑0.0036	↑0.0000	↑0.0000	n.s.0.4772	↑0.0000
b*	↑0.0000	↑0.0000	↑0.0001	↑0.0017	↑0.0000	↑0.0000	↑0.0000
C	↑0.0000	↑0.0000	↑0.0002	↑0.0018	↑0.0000	↑0.0000	↑0.0000
h^0^	↑0.0000	↑0.0000	↑0.0000	↑0.0000	↑0.0000	↑0.0008	↑0.0000
Hardness	↑0.0000	↑0.0000	↑0.0000	↑0.0059	↑0.0000	↑0.0063	↑0.0041
Cohesiveness	↑0.0000	↑0.0144	↑0.0000	n.s.0.1459	↑0.0000	n.s.0.6091	n.s.0.3344
Springiness	↑0.0000	n.s.0.1372	↑0.0433	↑0.0184	↑0.0003	n.s.0.6045	↑0.0282
Bacterial cell count	↑0.0000	↑0.0000	↑0.0000	↑0.0000	↑0.0000	↑0.0000	↑0.0000
Milky-creamy taste	n.s.0.5893	↑0.0259	↑0.0002	n.s.0.2530	↑0.0018	n.s.0.7884	n.s.0.9988
Sour taste	↑0.0000	↑0.0103	↑0.0000	n.s.0.3790	n.s.0.7685	n.s.0.8963	n.s.0.6830
Sweet taste	↑0.0166	↑0.0001	↑0.0000	n.s.0.5663	n.s.0.8543	n.s.0.8146	n.s.0.4515
Taste of the additive	n.s.0.1513	↑0.0017	↑0.0000	↑0.0006	↑0.0049	↑0.0000	n.s.0.7753
Off-taste	↑0.0001	↑0.0002	↑0.0000	↑0.0384	n.s.0.1807	n.s.0.1107	n.s.0.9891
Fermentation odor	n.s.0.1570	n.s.0.4774	n.s.0.4658	n.s.0.8571	n.s.0.9230	n.s.0.6640	n.s.0.9884
Odor of additives	n.s.0.4596	n.s.0.7174	↑0.0002	n.s.0.3737	n.s.0.9158	n.s.0.7707	n.s.0.8445
Off-odor	n.s.0.4393	n.s.0.4441	↑0.0013	↑0.0096	n.s.0.6507	n.s.0.9352	n.s.0.7159

Probiotic strains * Type of collagen = interaction ↑; Probiotic strains * Type of collagen = interaction ↑; Storage time * Type of collagen = interaction ↑; Probiotic strains * Storage time * Type of collagen = interaction ↑; *p* < 0.05 indicates significant effect; n.s.—no significant effect.

**Table 6:** Viable counts of probiotic bacteria [log cfu g^−1^] in fermented sheep’s milk.

Fermented Milk Group	Storage Time (Days)	
	1	21
LP	11.70 ^cB^ ± 0.75	9.40 ^aA^ ± 0.72
LP1.5W	9.71 ^abA^ ± 0.57	9.39 ^aA^ ± 0.90
LP3.0W	10.07 ^bA^ ± 0.27	9.74 ^aA^ ± 0.34
LP1.5H	11.14 ^cB^ ± 0.15	9.32 ^aA^ ± 0.51
LP3.0H	9.16 ^aA^ ± 0.57	9.17 ^aA^ ± 0.65
LR	11.13 ^cB^ ± 0.22	9.43 ^aA^ ± 0.97
LR1.5W	9.47 ^aA^ ± 0.40	9.23 ^aA^ ± 0.60
LR3.0W	10.83 ^bcB^ ± 0.45	9.55 ^aA^ ± 0.41
LR1.5H	10.20 ^bB^ ± 0.10	9.18 ^aA^ ± 0.54
LR3.0H	10.29 ^bB^ ± 0.51	9.44 ^aA^ ± 0.46
LC	8.99 ^aA^ ± 0.12	10.00 ^bB^ ± 0.75
LC1.5W	9.46 ^aA^ ± 0.15	9.18 ^aA^ ± 0.21
LC3.0W	9.14 ^aA^ ± 0.59	9.05 ^aA^ ± 0.23
LC1.5H	9.14 ^aA^ ± 0.14	10.09 ^bB^ ± 0.44
LC3.0H	9.23 ^aA^ ± 0.54	10.24 ^bB^ ± 0.45
LA	9.18 ^aA^ ± 0.53	9.11 ^aA^ ± 0.26
LA1.5W	9.36 ^aA^ ± 0.22	8.99 ^aA^ ± 0.86
LA3.0W	9.23 ^aA^ ± 0.30	9.18 ^aA^ ± 0.93
LA1.5H	9.40 ^aA^ ± 0.24	9.13 ^aA^ ± 0.60
LA3.0H	9.32 ^aA^ ± 0.21	9.12 ^aA^ ± 0.50

^A,B^—mean values denoted for one probiotic strain in storage time by different letters differ significantly at *p* ≤ 0.05; ^a–c^—mean values denoted for one probiotic strain in collagen type and dose given different letters differ significantly at *p* ≤ 0.05. Storage time: 1—after fermentation; 21—after 21 days; LP—control milk with *Lacticaseibacillus paracasei;* LP1.5W—milk with 1.5% collagen and *Lacticaseibacillus paracasei;* LP3.0W—milk with 3.0% collagen and *Lacticaseibacillus paracasei*; LP1.5H—milk with 1.5% collagen hydrolysate and *Lacticaseibacillus paracasei*; LP3.0H—milk with 3.0% collagen hydrolysate and *Lacticaseibacillus paracasei*; LR—control milk with *Lacticaseibacillus rhamnosus*; LR1.5W—milk with 1.5% collagen and *Lacticaseibacillus rhamnosus*; LR3.0W—milk with 3.0% collagen and *Lacticaseibacillus rhamnosus*; LR1.5H—milk with 1.5% collagen hydrolysate and *Lacticaseibacillus rhamnosus*; LR3.0H—milk with 3.0% collagen hydrolysate and *Lacticaseibacillus rhamnosus*.; LC—control milk with *Lacticaseibacillus casei*; LC1.5W—milk with 1.5% collagen and *Lacticaseibacillus casei*; LC3.0W—milk with 3.0% collagen and *Lacticaseibacillus casei*; LC1.5H—milk with 1.5% collagen hydrolysate and *Lacticaseibacillus casei*; LC3.0H—milk with 3.0% collagen hydrolysate and *Lacticaseibacillus casei*; LA—control milk with *Lactobacillus acidophilus*; LA1.5W—milk with 1.5% collagen and *Lactobacillus acidophilus*; LA3.0W—milk with 3.0% collagen and *Lactobacillus acidophilus*; LA1.5H—milk with 1.5% collagen hydrolysate and *Lactobacillus acidophilus*; LA3.0H—milk with 3.0% collagen hydrolysate and *Lactobacillus acidophilus*.

**Table 7:** Milk groups obtained in the experiment.

Bacterial Strain	Control Group, without Collagen	Type of Collagen	Group with 1.5% Collagen	Group with 3.0% Collagen
*Lacticaseibacillus casei*	LC	Collagen	LC1.5W	LC3.0W
		Collagen hydrolysate	LC1.5H	LC3.0H
*Lactobacillus acidophilus*	LA	Collagen	LA1.5W	LA3.0W
		Collagen hydrolysate	LA1.5H	LA3.0H
*Lacticaseibacillus paracasei*	LP	Collagen	LP1.5W	LP3.0W
		Collagen hydrolysate	LP1.5H	LP3.0H
*Lacticaseibacillus rhamnosus*	LR	Collagen	LR1.5W	LR3.0W
		Collagen hydrolysate	LR1.5H	LR3.0H

## Data Availability

Not applicable.
